# Effect of Saroglitazar on Glycemic Status: A Meta-analysis

**DOI:** 10.7759/cureus.99122

**Published:** 2025-12-13

**Authors:** Sandhiya Selvarajan, Anandabaskar Nishanthi, Ilanthamizhan Jayakumar, Shravan Venkatraman, Santhosh Shivabasappa, Jayaprakash Sahoo, Sadishkumar Kamalanathan, Melvin George

**Affiliations:** 1 Clinical Pharmacology, Jawaharlal Institute of Postgraduate Medical Education & Research, Puducherry, IND; 2 Pharmacology, Sri Manakula Vinayagar Medical College and Hospital, Puducherry, IND; 3 Pharmacology, Sri Ramachandra Institute of Higher Education and Research, Chennai, IND; 4 Clinical Pharmacology, Qure.ai, Bangalore, IND; 5 Endocrinology, Jawaharlal Institute of Postgraduate Medical Education & Research, Puducherry, IND; 6 Clinical Pharmacology, SRM Medical College Hospital & Research Centre, Chennai, IND; 7 Clinical Research, Hindu Mission Hospital, Chennai, IND

**Keywords:** diabetes type 2, diabetic dyslipidemia, glycosylated haemoglobin (hba1c), lipid-profile, saroglitazar

## Abstract

Aim: This systematic review and meta-analysis were conducted to assess the glycemic effect of saroglitazar in patients with type 2 diabetes or prediabetes and dyslipidemia.

Methods: PubMed and Google Scholar databases were searched for randomized controlled trials and observational studies till December 2023. The primary outcomes were changes in glycemic parameters such as glycated hemoglobin (HbA_1c_), fasting blood sugar (FBS), and post-prandial blood sugar (PPBS) levels, and the secondary outcomes included changes in lipid profile. The adverse drug reactions were noted and recorded. ROB2 was used for assessing the risk of bias.

Results: A total of 147 studies were screened, and 12 studies were included in the review. There was a significant reduction in FBS (-30.16 (95% CI: -40.36, -19.95) p<0.001, I^2^=98%), PPBS (-69.09 (95% CI: -85.72, -52.46) p<0.001, I^2^=96%), and HbA_1c_ (-0.93 (95% CI: -1.18, -0.67) p<0.001, I^2^=99%) levels following saroglitazar treatment. There was also a significant reduction in total cholesterol of -26.15 (95% CI: -42.44, -9.86) p=0.002, I^2^=98% and -58.25 (95% CI: -72.21, -44.30) p<0.001, I^2^=94% in the randomized controlled trials and observational studies, respectively. The study found a significant reduction in low-density lipoprotein and very-low-density lipoprotein levels, along with an improvement in high-density lipoprotein levels, in the saroglitazar group.

Conclusion: Our meta-analysis has found that saroglitazar causes a significant reduction in the HbA_1c_, FBS, and PPBS levels in patients treated for diabetic dyslipidemia.

## Introduction and background

Type 2 diabetes mellitus continues to rise at an alarming rate, with the prevalence growing more than four times in the last three decades. Nearly 800 million people were estimated to be living with diabetes as of 2022 [1]. It remains one of the strongest risk factors for life-threatening illnesses such as coronary artery disease, stroke, chronic kidney disease, and cancer. Drugs such as metformin, sulfonylureas, pioglitazone, and voglibose have helped millions of patients with diabetes to achieve substantial glycemic control. Nevertheless, a considerable proportion of patients do not achieve adequate control of their blood sugars in spite of being on multiple drugs, leaving insulin as a possible option for them [2]. Saroglitazar, a dual peroxisome proliferator-activated receptor (PPAR) α/γ agonist, has been approved for the treatment of diabetic dyslipidemia by the Indian drug regulatory authority [3,4].

Saroglitazar has an advantage over hypoglycemic drugs, especially in not causing weight gain. The PRESS XII study conducted in India across 39 centers demonstrated that saroglitazar 2 mg and 4 mg could reduce glycated hemoglobin (HbA_1c_) by -1.38 ± 1.99 and by -1.47 ± 1.92, respectively [5]. A meta-analysis by Dutta et al. revealed that saroglitazar had a marked reduction of triglyceride and low-density lipoprotein (LDL) levels. However, their study did not show any improvement in HbA_1c_ and high-density lipoprotein (HDL) levels [6]. Since the literature on the effect of saroglitazar on glycemic status and lipid profile is scarce and contradictory [7,8], this meta-analysis was conducted to evaluate the changes in glycemic status, lipid profile, and adverse drug reactions with saroglitazar in patients with type 2 diabetes or prediabetes and dyslipidemia.

## Review

Methods

Search Strategy and Selection Criteria

The study protocol was registered with the Prospective Register of Systematic Reviews (PROSPERO) under the ID CRD42020197698, and was performed and reported as per the Preferred Reporting Items for Systematic reviews and Meta-Analysis (PRISMA) guidelines [9]. Randomized controlled trials and observational studies were included if they assessed saroglitazar as an add-on therapy in patients aged ≥18 years with type 2 diabetes mellitus or prediabetes and dyslipidemia, and reported fasting or post-prandial blood sugar (PPBS) or HbA_1c_ as one of the outcome parameters. The exclusion criteria included conference abstracts and studies involving patients with diabetes or prediabetes and other comorbidities.

A comprehensive and systematic search was performed in PubMed and Google Scholar, from inception to December 2023. We used the MeSH terms and keywords related to “Saroglitazar”, “prediabetes”, “type 2 diabetes mellitus”, and “diabetic dyslipidemia.” In addition, all the references of the included articles were scrutinized, and a snowball search through Google was performed to identify any additional relevant articles.

Study selection and data extraction

The titles and/or abstracts of studies were retrieved using the search strategy, and those from additional sources were screened independently by two review authors to identify studies that potentially met the inclusion and exclusion criteria outlined above. The full text of these potentially eligible studies was retrieved and independently assessed for eligibility by two review team members. Any disagreement between them over the eligibility of particular studies was resolved through discussion with the third reviewer. A standardized, pre-formatted form was used to extract data from the included studies for assessment of study quality and evidence synthesis. Extracted information included: study setting, study population, participant demographics, and baseline characteristics; details of the intervention and control conditions; study methodology; recruitment, outcomes, and times of measurement; and information for assessment of the risk of bias. Glycemic parameters such as HbA_1c_, fasting blood sugar (FBS), and PPBS levels and lipid parameters such as serum total cholesterol, serum triglyceride, LDL, HDL, very-low density lipoprotein (VLDL), and non-HDL levels were collected with adverse drug reactions. Two review authors independently extracted the data, and the discrepancies identified were resolved through discussion (with the third author where necessary).

Risk of bias and quality assessment

Two review authors independently assessed the risk of bias among the included observational studies and clinical trials. Quality assessment for case-control/cohort studies was performed using the Newcastle-Ottawa quality assessment scale (Table 1) [10].

**Table 1:** Quality assessment for case-control/cohort studies using the Newcastle-Ottawa quality assessment scale

Authors	Selection				Comparability	Outcome			Total score
	Representativeness of the exposed cohort	Selection of the non-exposed cohort	Ascertainment of exposure	Outcome not present at the start of the study	Control for age, sex, comorbidities, and severity measure	Assessment of outcomes	Length of follow-up	Adequacy of follow-up	
Baidya et al. [11]	*	*	*	*	*	*	*	*	8
Bage et al. [12]	*	*	*	*	*	*	*	*	8
Goyal et al. [13]	*	*	*	*	*	*	*		7
Mohit et al. [14]	*	*	*	*	*	*	*	*	8
Shetty et al. [15]	*	*	*	*	*	*	*		7

ROB2 assessment was performed to check the quality of clinical trials (Table 2) [16].

**Table 2:** ROB2 assessment for checking the quality of clinical trials

+	Low risk
!	Some concerns
-	High risk

D1	Randomization process
D2	Deviations from the intended interventions
D3	Missing outcome data
D4	Measurement of the outcome
D5	Selection of the reported result

Study ID	D1	D2	D3	D4	D5	Overall
Jain et al. [17]	+	+	-	+	+	-
Jani et al. [18]	+	+	+	+	+	+
Krishnappa et al. [5]	+	+	+	+	+	+
Pai et al. [19]	+	+	!	+	+	!
Rastogi et al. [20]	+	+	-	+	+	-
Gahlot et al. [21]	-	+	+	-	+	+
Bhosle et al. [22]	-	+	+	-	+	-

Data synthesis and statistical analysis

The extracted data were transferred into the Review Manager (RevMan) version 5.4 (The Cochrane Collaboration, London, England, UK) software. Data were synthesized using the random-effect model. Pooled mean differences with 95% CIs were estimated for the outcomes of HbA_1c_, FBS, PPBS, and lipid profile for every included study arm. Forest plots were constructed using the changes in continuous variable outcomes calculated. I^2^ statistics were used to assess the heterogeneity among the studies. A subgroup analysis was performed based on the type of study (randomized controlled trial or observational) and the dose of saroglitazar (2 mg or 4 mg). Publication bias was assessed by visually inspecting the funnel plots. No evidence of publication bias was detected in the study.

Results

**Figure 1:**
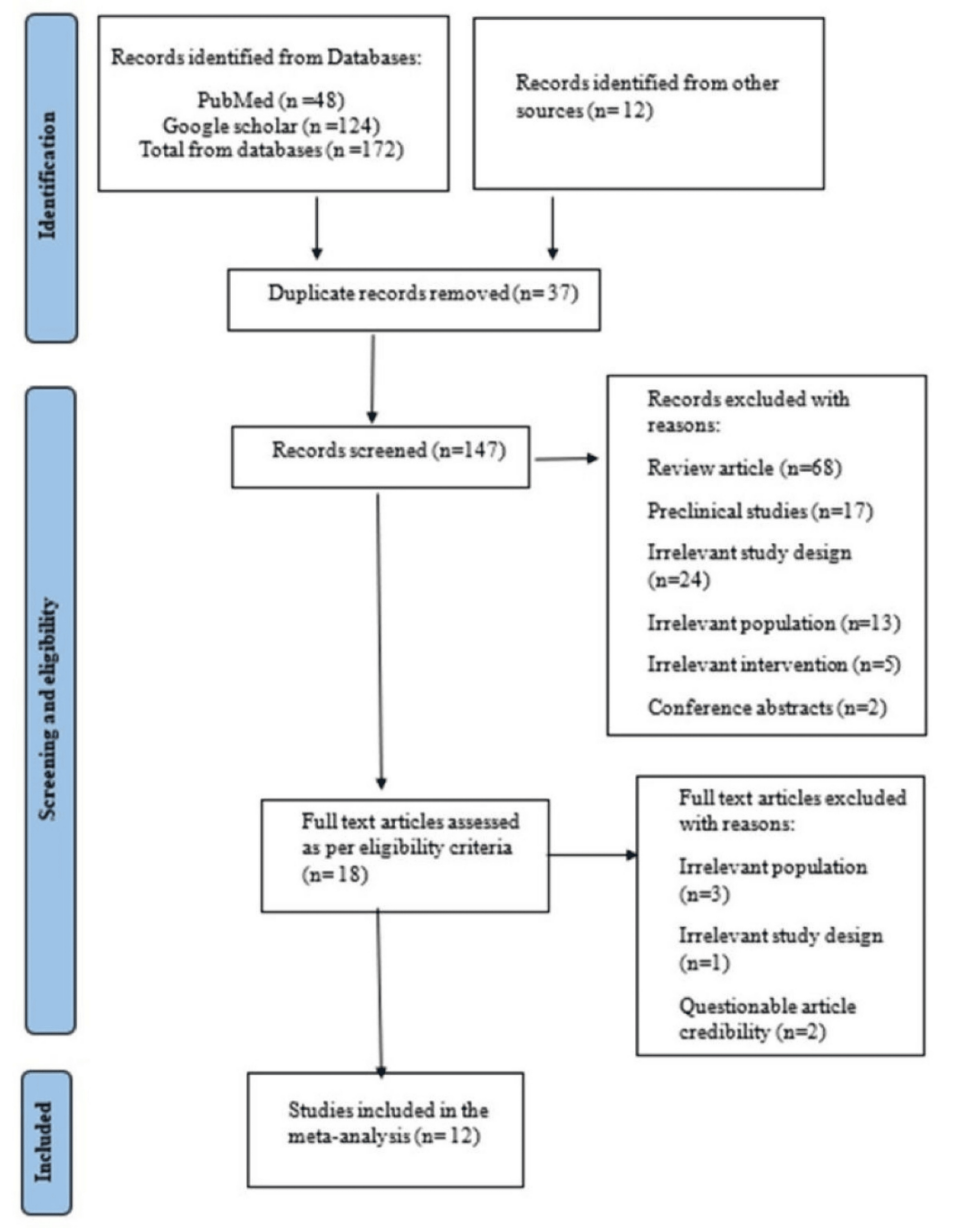
PRISMA flow diagram of the study selection process PRISMA: Preferred Reporting Items for Systematic reviews and Meta-Analysis

A total of 147 studies were screened, and 12 studies were included in the meta-analysis as per the eligibility criteria (Figure 1).

Out of the included 12 studies, 5 were observational studies. Information regarding the demographic and baseline characteristics of both interventional and observational studies is shown in Tables 3, 4, respectively.

**Table 3:** Demographics and baseline characteristics: Interventional studies *For RCT; **denotes mean change from baseline. In Jain et al., the levels of HbA1c were measured using an automated HPLC-based system using an ion-exchange cartridge (D-10, Bio-Rad Laboratories, Inc., Hercules, CA, USA) [17]. In Rastogi et al., the levels of HbA1c were measured using an automatic analyzer (D10—Bio-Rad Laboratories Inc., USA) [20]. BMI: Body mass index; BP: Blood pressure; HbA1c: Glycated hemoglobin; n: Sample size; NM: Not mentioned; RCT: Randomized controlled trial; SD: Standard deviation.

Study no.	Author, year	Site	Disease condition	Intervention (n)	Study type (sequence generation*/allocation concealment*/blinding*)	Duration of treatment (days)	Co-medications	Age (years), mean ± SD	Female sex, n (%)	BMI (kg/m^2^), mean ± SD	Baseline systolic BP, mean ± SD	Baseline diastolic BP, mean ± SD
1	Bhosle et al., 2018 [22]	Maharashtra, India	Prediabetes with dyslipidemia	Saroglitazar 4 mg (40)	Interventional (single arm)	168	Antihypertensives	48.15 ± 7.53	12 (30)	26.93 ± 2.26	NM	NM
2	Gahlot et al., 2023 [21]	Delhi, India	Type 2 diabetes with dyslipidemia	Saroglitazar 40 mg (20)	RCT, open-label study	84	Atorvastatin	46.55 ± 7.74	17 (85)	26.1 ± 3.62	NM	NM
				Fenofibrate 200 mg (20)				48.25 ± 7.12	15 (75)	26.4 ± 3.12	NM	NM
3	Pai et al., 2014 [19]	Multicentric (14 sites in India)	Type 2 Diabetes with dyslipidemia	Saroglitazar 2 mg (41)	RCT (block randomization/sealed opaque envelope/double-blinded)	168	Metformin, sulfonylurea	48.9 ± 8.98	15 (36.6)	26.5 ± 3.63	129 ± 8.7	NM
				Saroglitazar 4 mg (41)				47.3 ± 9.10	17 (41.5)	27.5 ± 3.90	129 ± 8.2	81 ± 5.5
				Pioglitazone 45 mg (40)				49.9 ± 10.98	16 (40)	27.0 ± 3.72	126 ± 8.1	81 ± 4.9
4	Jani et al., 2014 [18]	Multicentric (29 centers in India)	Type 2 diabetes with hypertriglyceridemia	Saroglitazar 2 mg (101)	RCT (block randomization/sealed opaque envelope/double-blinded)	84	Atorvastatin	50.4 ± 9.01	39 (39)	27.1 ± 3.56	131 ± 9.7	83 ± 5.9
				Saroglitazar 4 mg (99)				51.2 ± 8.66	43 (43.4)	26.8 ± 3.37	131 ± 9.9	83 ± 6.0
				Placebo (102)				49.8 ± 9.95	47 (46.1)	27.0 ± 3.79	131 ± 10.3	83 ± 5.7
5	Jain et al., 2019 [17]	Chandigarh, India	Type 2 diabetes with hypertriglyceridemia	Saroglitazar 4 mg (15)	RCT (block randomization/sealed tamper-proof envelope/double-blinded)	112	Glimepiride (used as rescue medication)	40.9 ± 9.6	0	27.3 ± 2.5	NM	NM
				Placebo (15)				47 ± 8.8	3 (20)	27.8 ± 2.5	NM	NM
6	Rastogi et al., 2020 [20]	Chandigarh and Mumbai, India	Type 2 diabetes with dyslipidemia	Saroglitazar 4 mg (15)	RCT (block randomization/sealed opaque envelope/double-blinded)	84	Metformin, sitagliptin, vildagliptin, and glimepiride	53.1 ± 8.8	8 (53)	27.3 ± 2.7	121.8 ± 4.1	81.7 ± 3.1
				Placebo (15)				54.9 ± 7.8	6 (40)	28.9 ± 2.8	120.4 ± 3.9	82.0 ± 4.6
7	Krishnappa et al., 2020 [5]	Multicentric (39 sites in India)	Type 2 diabetes	Saroglitazar 2 mg (380)	RCT (SAS® statistical software (version: 9.4; SAS Institute Inc., USA) generated randomization/scratch card system/double-blinded)	168	Metformin	51.90 ± 10.38	164 (43.16)	26.48 ± 4.03	−1.49 ± 9.08**	−0.40 ± 7.00**
				Saroglitazar 4 mg (386)				51.34 ± 10.06	143 (37.05)	25.94 ± 3.87	−1.41 ± 9.73 **	NM
				Pioglitazone 30 mg (389)				51.84 ± 9.76	167 (42.93)	26.33 ± 4.07	−1.95 ± 9.80**	−0.40 ± 6.91 **

**Table 4:** Demographics and baseline characteristics: Observational studies In Bage et al., the levels of HbA1c were measured using an automatic analyzer (D10—Bio-Rad) [12]. BMI: Body mass index; BP: Blood pressure; CAD: Coronary artery disease; DPP4: Dipeptidyl peptidase-4; GLP-1: Glucagon-like peptide-1; HbA1c: Glycated hemoglobin; n: Sample size; NM: Not mentioned; SD: Standard deviation.

Study no.	Author, year	Site	Disease condition	Dose of saroglitazar (mg)	Duration of treatment (days)	Co-medications	Age (years), mean ± SD	Female sex, n (%)	BMI (kg/m^2^), mean ± SD	Baseline systolic BP, mean ± SD	Baseline diastolic BP, mean ± SD
1	Goyal et al., 2019 [13]	Rajasthan, India	Type 2 diabetes with dyslipidemia	4	84	Metformin, sulfonylurea, insulin, DPP4 inhibitors, and statin	Age distribution: 40-49 (42%), 50-59 (30%), and ≥60 (10%)	NM	26.63 ± 1.3	146 ± 7.4	88.16 ± 5.7
2	Shetty et al., 2015 [15]	Multicentric, India	Type 2 diabetes with dyslipidemia	4	84	Statin (atorvastatin, rosuvastatin, pitavastatin, simvastatin, and pravastatin), metformin, sulfonylurea, gliptins, alpha-glucosidase inhibitors, insulin, thiazolidinediones, meglitinide analogs, GLP-1 agonist, and bromocriptine	53 ± 10	1175 (37.5)	27.0 ± 4.17	NM	NM
3	Bage et al., 2023 [12]	Puducherry, India	Type 2 diabetes with dyslipidemia	4	84	Antidiabetic drugs and statin	48.01 ± 5.73	36(66.6)	27.13 ± 4.10	136.35 ± 21.87	84.06 ± 11.96
4	Mohit et al., 2017 [14]	multicentric, India	Type 2 diabetes with dyslipidemia with or without CAD (we have included only without CAD for glycemic and lipid parameters)	4	168	Antidiabetic drugs and statin	58.58 ±14 (for n=50, including CAD patients)	21 (42) (for n=50, including CAD patients)	Not mentioned but mean ± SD weight = 74.16 ± 13.6 (for n=50, including CAD patients)	NM	NM
5	Baidya et al., 2021 [11]	Kolkata, India	Type 2 diabetes with dyslipidemia	4	84	Antidiabetic drugs and statin	57.6 ± 8.1	58 (38.7)	26.8 ± 3.89	NM	NM

**Figure 2:**
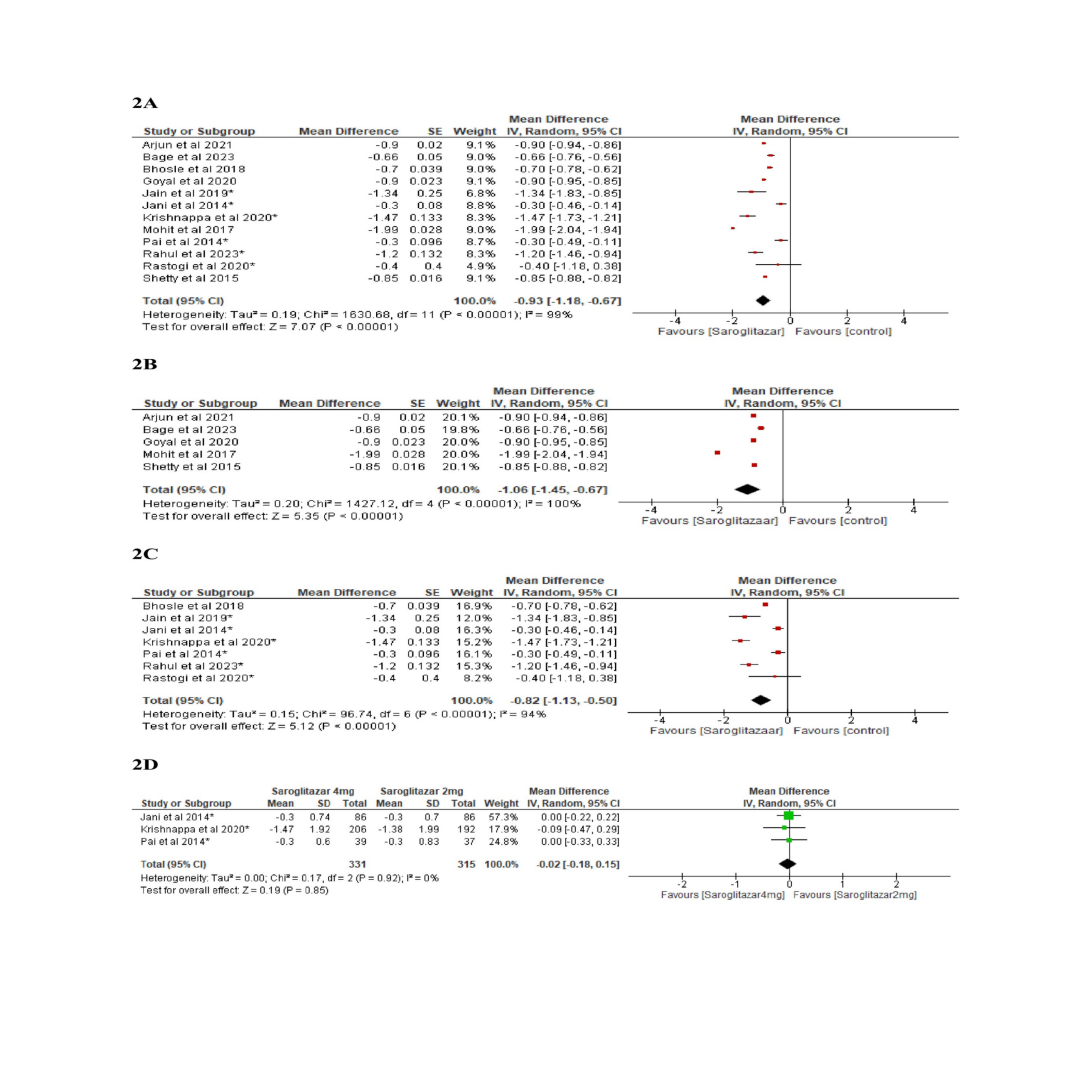
Effect of saroglitazar on HbA1C levels Figure 2a: Overall (observational studies and RCTs) [5,11-15,17-22] Figure 2b: Observational studies [11-15] Figure 2c: RCTs comparing saroglitazar vs. placebo [5,17-22] Figure 2d: Saroglitazar 2 mg vs. 4 mg [5,18,19] HbA1c: Glycated hemoglobin; RCT: Randomized controlled trial.

**Figure 3:**
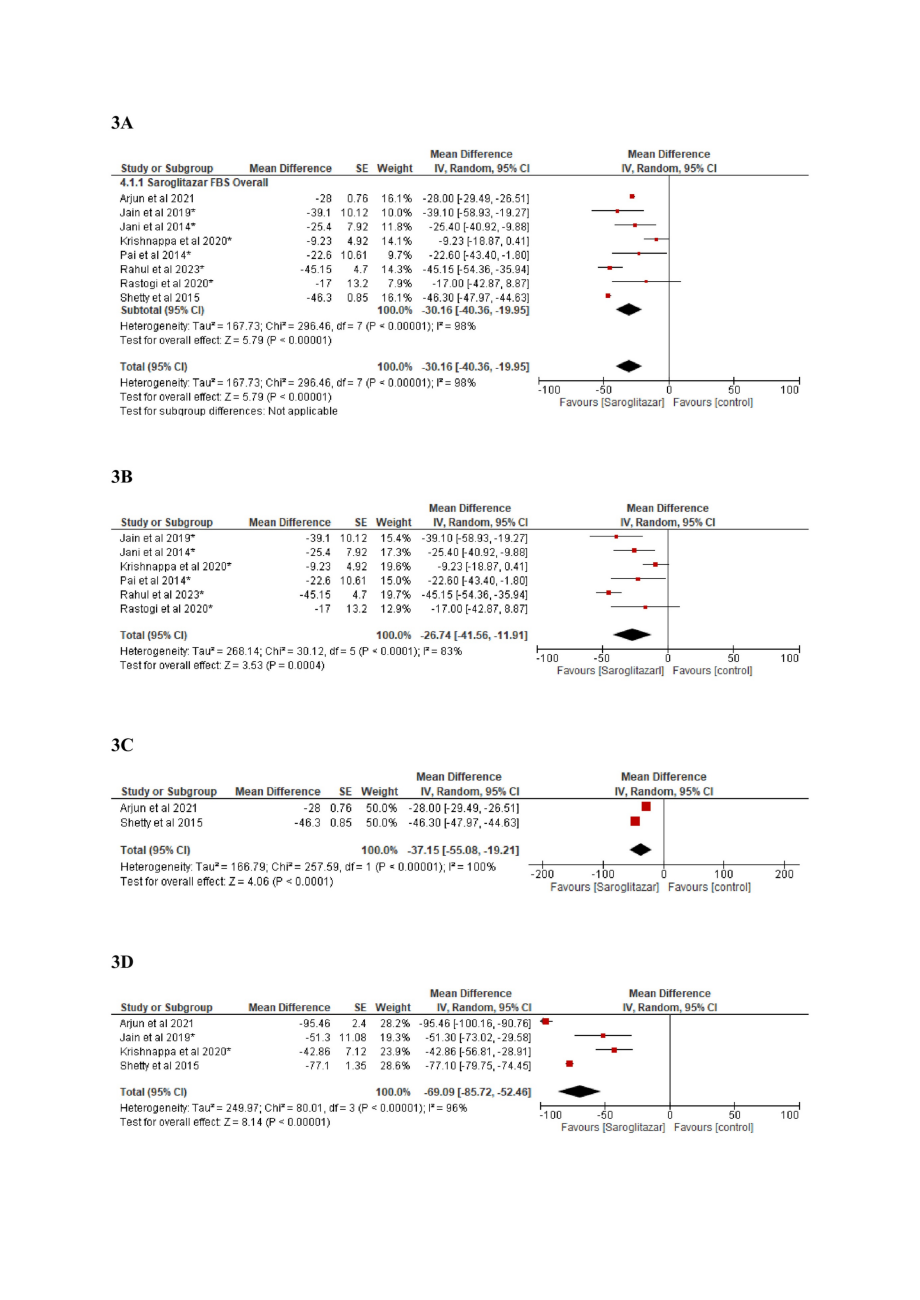
Effect of saroglitazar on FBS and PPBS levels Figure 3a: Effect of saroglitazar on FBS (overall) [5,11,15,17-21] Figure 3b: Effect of saroglitazar on FBS (randomized controlled trials) [5,17-21] Figure 3c: Effect of saroglitazar on FBS (observational) [11,15] Figure 3d: Effect of saroglitazar on PPBS (overall) [5,11,15,17] FBS: Fasting blood sugar; PPBS: Post-prandial blood sugar.

As compared to the control group, treatment with saroglitazar led to a significant reduction in HbA_1c_ levels (-0.93 (95% CI: -1.18, -0.67) p<0.001, I^2^=99%) (Figure 2). There was a significant mean reduction in FBS (-30.16 (95% CI: -40.36, -19.95) p<0.001, I^2^=98%) and PPBS (-69.09 (95% CI: -85.72, -52.46) p<0.001, I^2^ =96%) levels (Figure 3). There was no difference in HbA_1c_ reduction between 2 mg and 4 mg doses of saroglitazar.

The glycemic and lipid parameters of both interventional and observational studies are shown in Tables 5, 6, respectively.

**Table 5:** Glycemic and lipid parameters: Interventional studies *Expressed as LSM ± SD; **expressed as mean ± SED. A: After intervention; B: Before intervention; C: Absolute change from baseline; FBS: Fasting blood sugar; HbA1c: Glycated hemoglobin; HDL: High-density lipoprotein; LDL: Low-density lipoprotein; LSM: Least squares mean; NM: Not mentioned; SD: Standard deviation; SED: Standard error of difference; VLDL: Very-low-density lipoprotein.

Study no.	Author, year	Intervention (n)		HbA_1c_ (%), mean ± SD	FBS (mg/dL), mean ± SD	PPBS (mg/dL), mean ± SD	Serum total cholesterol (mg/dL), mean ± SD	Serum triglyceride (mg/dL), mean ± SD	LDL (mg/dL), mean ± SD	HDL (mg/dL), mean ± SD	VLDL (mg/dL), mean ± SD	Non-HDL (mg/dL), mean ± SD
1	Bhosle et al., 2018 [22]	Saroglitazar 4 mg (40)	B	6.3 ± 0.16	NM	NM	324.7 ± 43.39	348 ± 86.98	209.8 ± 47.67	44.8 ± 5.71	NM	278.6 ± 42.38
			A	5.5 ± 0.30	NM	NM	270.1 ± 43.61	216.4 ± 72.34	177.9 ± 47.56	49.0 ± 6.13	NM	224.1 ± 47.15
2	Pai et al., 2014 [19]	Saroglitazar 2 mg (41)	B	8.1 ± 0.86	143.9 ± 42.35	NM	202.4 ± 47.60	253.9 ± 68.44	134.8 ± 42.56	36.8 ± 12.09	50.3 ± 14.17	NM
			A	NM	NM	NM	NM	NM	NM	NM	NM	NM
			C	–0.3 ± 0.83*	–11.3 ± 50.11*	NM	2.5 ± 43.49*	–78.2 ± 81.98*	3.6 ± 40.07*	2.8 ± 11.27*	–15.2 ± 16.86*	NM
		Saroglitazar 4 mg (41)	B	7.9 ± 0.58	152.7 ± 65.99	NM	197.3 ± 40.98	257.0 ± 52.39	130.8 ± 38.83	35.3 ± 9.64	52.4 ± 12.35	NM
			A	NM	NM	NM	NM	NM	NM	NM	NM	NM
			C	–0.3 ± 0.60*	–22.6 ± 66.30*	NM	–18.5 ± 40.62*	–115.4 ± 68.11*	–12.0 ± 39.38*	0.2 ± 7.78*	–23.9 ± 15.26*	NM
		Pioglitazone 45 mg (40)	B	8.2 ± 0.75	138.2 ± 31.94	NM	185.8 ± 29.91	265.0 ± 61.66	116.6 ± 29.25	38.3 ± 10.85	55.1 ± 18.78	NM
			A	NM	NM	NM	NM	NM	NM	NM	NM	NM
			C	–0.4 ± 0.72*	–21.8 ± 46.24*	NM	9.1 ± 28.77*	–33.3 ± 162.41*	3.5 ± 23.17*	2.0 ± 6.86*	–8.8 ± 24.81*	NM
3	Jani et al., 2014 [18]	Saroglitazar 2 mg (101)	B	8.9 ± 1.84	179.6 ± 71.23	NM	200.6 ± 38.11	273.3 ± 78.58	132.5 ± 30.43	36.6 ± 8.45	NM	NM
			A	NM	NM	NM	NM	NM	NM	NM	NM	NM
			C	−0.3 ± 0.08**	−23.6 ± 7.92**	NM	−48.7 ± 3.54**	−132.7 ± 8.30 **	−40.1 ± 3.01**	2.5 ± 0.89 **	−23.3 ± 2.03**	−51.4 ± 3.59**
		Saroglitazar 4 mg (n= 99)	B	8.9 ± 1.77	176.3 ± 71.58	NM	210.4 ± 37.20	287.3 ± 85.94	140.2 ± 29.36	39.1 ± 11.19	NM	NM
			A	NM	NM	NM	NM	NM	NM	NM	NM	NM
			C	−0.3 ± 0.08**	−25.4 ± 7.92**	NM	−56.4 ± 3.53**	−139.5 ± 8.29**	−45.5 ± 3.00**	1.3 ± 0.89 **	−27.2 ± 2.02**	−57.7 ± 3.58**
		Placebo (102)	B	9.2 ± 1.81	184.1 ± 68.27	NM	209.5 ± 39.31	286.6 ± 78.92	140.1 ± 33.58	38.5 ± 12.06	NM	NM
			A	NM	NM	NM	NM	NM	NM	NM	NM	NM
			C	−0.2 ± 0.07**	−2.0 ± 7.58**	NM	−40.3 ± 3.38**	−78.0 ± 7.93**	−35.6 ± 2.88 **	−1.6 ± 0.85**	−15.0 ± 1.94**	−38.6 ± 3.43**
4	Jain et al., 2019 [17]	Saroglitazar 4 mg (15)	B	8 ± 0.7	154.9 ± 23.7	240.73 ± 40.1	192.4 ± 42.9	325.6 ± 129.3	116.4 ± 36.3	37.49 ± 9.6	NM	NM
			A	6.7 ± 1	115.8 ± 28.2	189.4 ± 47.5	178.9 ± 40.2	209.4 ± 124.4	112.6 ± 31	42.95 ± 10.6	NM	NM
		Placebo (15)	B	7.7 ± 0.6	141.3 ± 16.2	239.4 ± 41.6	217.6 ± 45.5	236.3 ± 83.1	146.7 ± 45.3	45.3 ± 8.5	NM	NM
			A	7.2 ± 1	132 ± 24.2	214.4 ± 35.8	189.2 ± 49.9	245.5 ± 109.1	119.4 ± 44.2	41 ± 7.9	NM	NM
5	Rastogi et al., 2020 [20]	Saroglitazar 4 mg (15)	B	8.0 ± 1.0	150.8 ± 43.9	NM	151.4 ± 36.4	Post-clearance value = 154.4 ± 79.6	89.0 ± 36.3	47.4 ± 8.8	NM	104.2 ± 35.1
			A	7.6 ± 1.9	130.3 ± 46.9	NM	151.8 ± 30.7	Post-clearance value = 136.8 ± 60.6	88.2 ± 31.4	45.9 ± 10.5	NM	105.9 ± 31.3
		Placebo (15)	B	7.6 ± 0.9	141.0 ± 28.5	NM	176.7 ± 41.4	Post-clearance value = 187.8 ± 61.5	117.4 ± 38.4	37.7 ± 7.6	NM	139.2 ± 42.9
			A	8.8 ± 1.4	166.3 ± 33.5	NM	171.5 ± 36.1	Post-clearance value = 184.8 ± 87.0	112.9 ± 31.8	42.4 ± 10.2	NM	129.3 ± 38.5
6	Krishnappa et al., 2020 [5]	Saroglitazar 2 mg (380)	B	9.76 ± 1.59	166.08 ± 46.14	275.90 ± 84.74	176.98 ± 42.67	163.87 ± 91.49	117.11 ± 36.92	42.39 ± 10.58	32.77 ± 18.30	134.62 ± 41.06
			A	NM	NM	NM	NM	NM	NM	NM	NM	NM
			C	−1.38 ± 1.99	−0.09 ± 72.72	−35.46 ± 108.81	−6.31 ± 48.48	−17.20 ± 125.30	−10.11 ± 42.06	2.23 ± 12.83	−3.44 ± 25.06	−8.57 ± 46.30
		Saroglitazar 4 mg (386)	B	9.72 ± 1.58	165.41 ± 51.39	277.42 ± 90.57	174.03 ± 39.32	172.52 ± 123.67	112.93 ± 34.89	41.50 ± 10.47	34.50 ± 24.73	132.54 ± 39.12
			A	NM	NM	NM	NM	NM	NM	NM	NM	NM
			C	−1.47 ± 1.92	−8.09 ± 78.76	−44.36 ± 103.73	−12.67 ± 42.22	−40.09 ± 144.91	−12.49 ± 38.99	0.92 ± 10.69	−8.02 ± 28.98	−13.61 ± 41.83
		Pioglitazone 30 mg (389)	B	9.49 ± 1.54	165.08 ± 51.45	277.35 ± 88.05	176.42 ± 37.83	166.20 ± 89.93	116.77 ± 32.31	42.64 ± 12.72	33.24 ± 17.99	133.78 ± 35.39
			A	NM	NM	NM	NM	NM	NM	NM	NM	NM
			C	−1.41 ± 1.86	−12.70 ± 67.98	−45.52 ± 101.73	−1.28 ± 44.84	−18.81 ± 99.43	−5.60 ± 37.84	2.11 ± 13.65	−3.76 ± 19.89	−3.42 ± 44.69
7	Gahlot et al., 2023 [21]	Saroglitazar 40 mg (20)	B	8.01 ± 0.57	155.90 ± 22.32	NM	207.85 ± 23.13	284.75 ± 50.05	103.05 ± 33.42	34.15 ± 6.23	51.90 ± 6.77	NM
			A	NM	NM	NM	NM	NM	NM	NM	NM	NM
			C	1.20 ± 0.59	45.15 ± 21.02	NM	57.75 ± 21.18	125.80 ± 45.70	35.90 ± 13.76	11.25 ± 6.68	13.45 ± 6.76	NM
		Fenofibrate 200 mg (20)	B	7.93 ± 0.57	140.65 ± 27.14	NM	213.50 ± 17.67	283.60 ± 46.11	100.90 ± 33.48	34.10 ± 5.78	52.45 ± 7.31	NM
			A	NM	NM	NM	NM	NM	NM	NM	NM	NM
			C	0.26 ± 0.45	26.95 ± 23.18	NM	67.95 ± 14.25	108.65 ± 28.25	42.50 ± 16.87	9.25 ± 4.24	14.60 ± 6.89	NM

**Table 6:** Glycemic and lipid parameters: Observational studies *Expressed as LSM ± SD; **expressed as mean ± SED. A: After intervention; B: Before intervention; C: Absolute change from baseline; FBS: Fasting blood sugar; HbA1c: Glycated hemoglobin; HDL: High-density lipoprotein; LDL: Low-density lipoprotein; LSM: Least squares mean; NM: Not mentioned; SD: Standard deviation; SED: Standard error of difference; VLDL: Very-low-density lipoprotein.

Study no.	Author, year	Duration of treatment with saroglitazar 4 mg (days)	Measurement (B/A)	HbA_1c_ (%), mean ± SD	FBS (mg/dL), mean ± SD	PPBS (mg/dL), mean ± SD	Serum total cholesterol (mg/dL), mean ± SD	Serum triglyceride (mg/dL), mean ± SD	LDL (mg/dL), mean ± SD	HDL (mg/dL), mean ± SD	VLDL (mg/dL) mean ± SD	Non-HDL (mg/dL), mean ± SD
1	Goyal et al., 2019 [13]	84	B	7.80	149.5 ± 37.25	230.9 ± 62.02	215 ± 33.6	318 ± 82.3	125.9	38.72	50.42	176.4
			A	6.75	105.9	161	163.7 ± 29.25	193.3 ± 57.32	90.48	38.62	35.28	133.7
2	Shetty et al., 2015 [15]	84	B	8.3 ± 1.28	175.2 ± 53.34	262.4 ± 80.26	240.2 ± 63.04	312.3 ± 122.65	139.5 ± 42.16	38.8 ± 8.65	52.0 ± 9.95	201.8 ± 64.08
			A	7.4 ± 0.89	128.9 ± 33.17	185.2 ± 48.25	189.9 ± 41.29	188.7 ± 61.40	112.4 ± 30.83	41.0 ± 7.14	34.8 ± 9.07	149.4 ± 41.02
3	Bage et al., 2023 [12]	84	B	8.54 ± 0.57	NM	NM	198.77 ± 46.46	292.33 ± 80.86	126.90 ± 32.31	NM	NM	155.44 ± 27.05
			A	7.88 ± 0.53	NM	NM	166.14 ± 53.90	184.46 ± 91.09	106.77 ± 34.09	NM	NM	125.30 ± 28.76
4	Mohit et al., 2017 [14]	168	C	–1.99 ± 0.14**	NM	NM	–89.24 ± 7.61**	–106.48 ± 11.47**	–84.04 ± 4.01**	1.16 ± 3.24**	NM	NM
5	Baidya et al., 2021 [11]	84	B	8.02 ± 0.3	160.52 ± 7.23	132.47 ± 5.81	310.2 ± 33.04	669.93 ± 81.22	167.68 ± 10.881	40.42 ± 5.87	NM	270.8 ± 34.08
			A	7.71 ± 0.5	269.62 ± 24.39	174.16 ± 16.31	240.7 ± 23.41	268.72 ± 82.32	118.88 ± 12.16	41.16 ± 6.13	NM	198.6 ± 28.02

The pooled analysis of interventional and observational studies found that there was a mean reduction in total cholesterol of -26.15 (95% CI: -42.44, -9.86) p=0.002, I^2^=98% and -58.25 (95% CI: -72.21, - 44.30) p<0.001, I^2^=94%, respectively.

**Figure 4:**
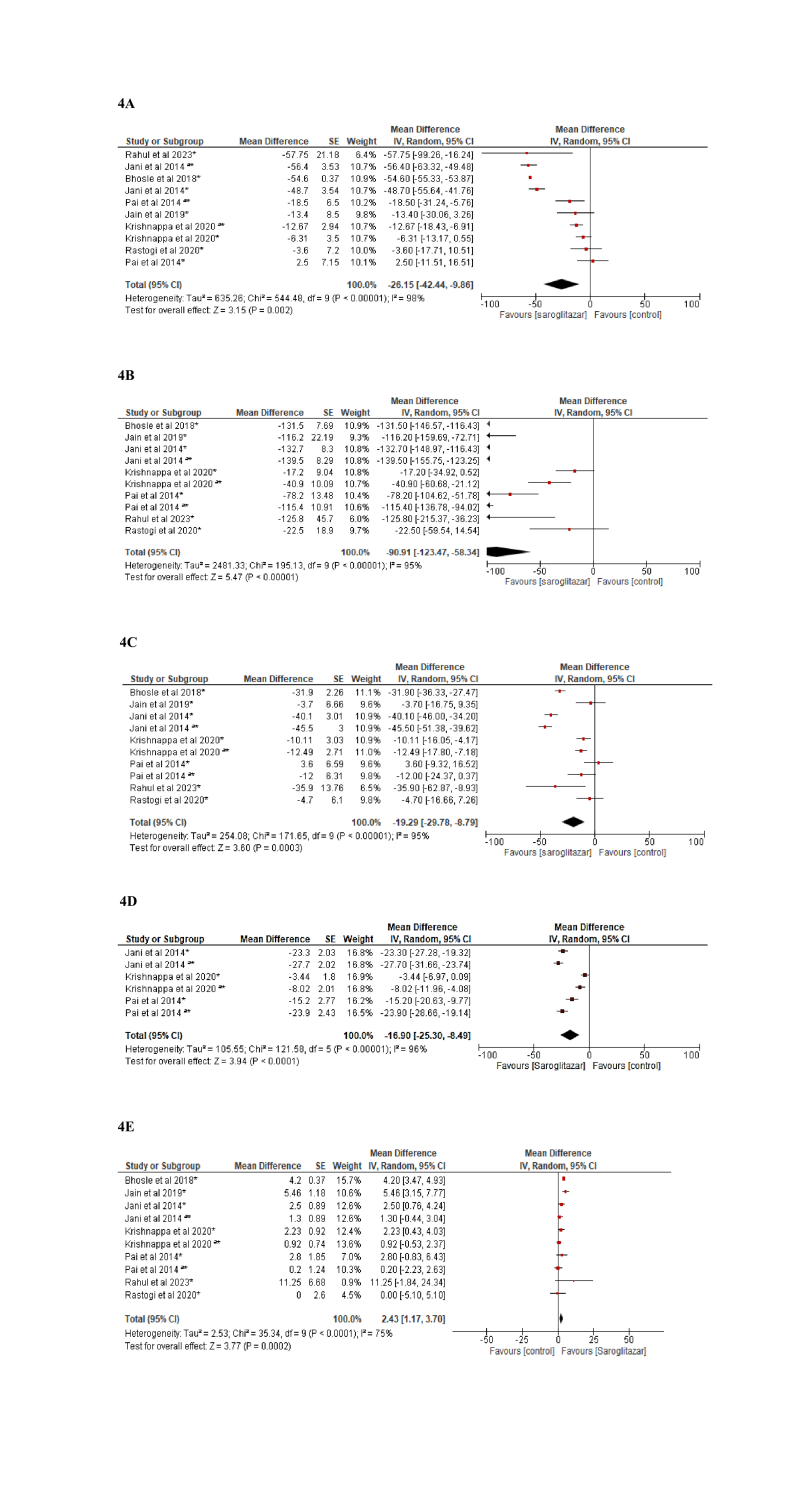
Forest plot showing reduction in the lipid levels with saroglitazar 2 mg or 4 mg for the treatment of diabetic dyslipidemia (randomized controlled trials) Figure 4a: Total cholesterol [5,17-22] Figure 4b: Triglycerides [5,17-22] Figure 4c: LDL [5,17-22] Figure 4d: VLDL [5,18,19] Figure 4e: HDL [5,17-22] HDL: High-density lipoprotein; LDL: Low-density lipoprotein; VLDL: Very-low-density lipoprotein.

**Figure 5:**
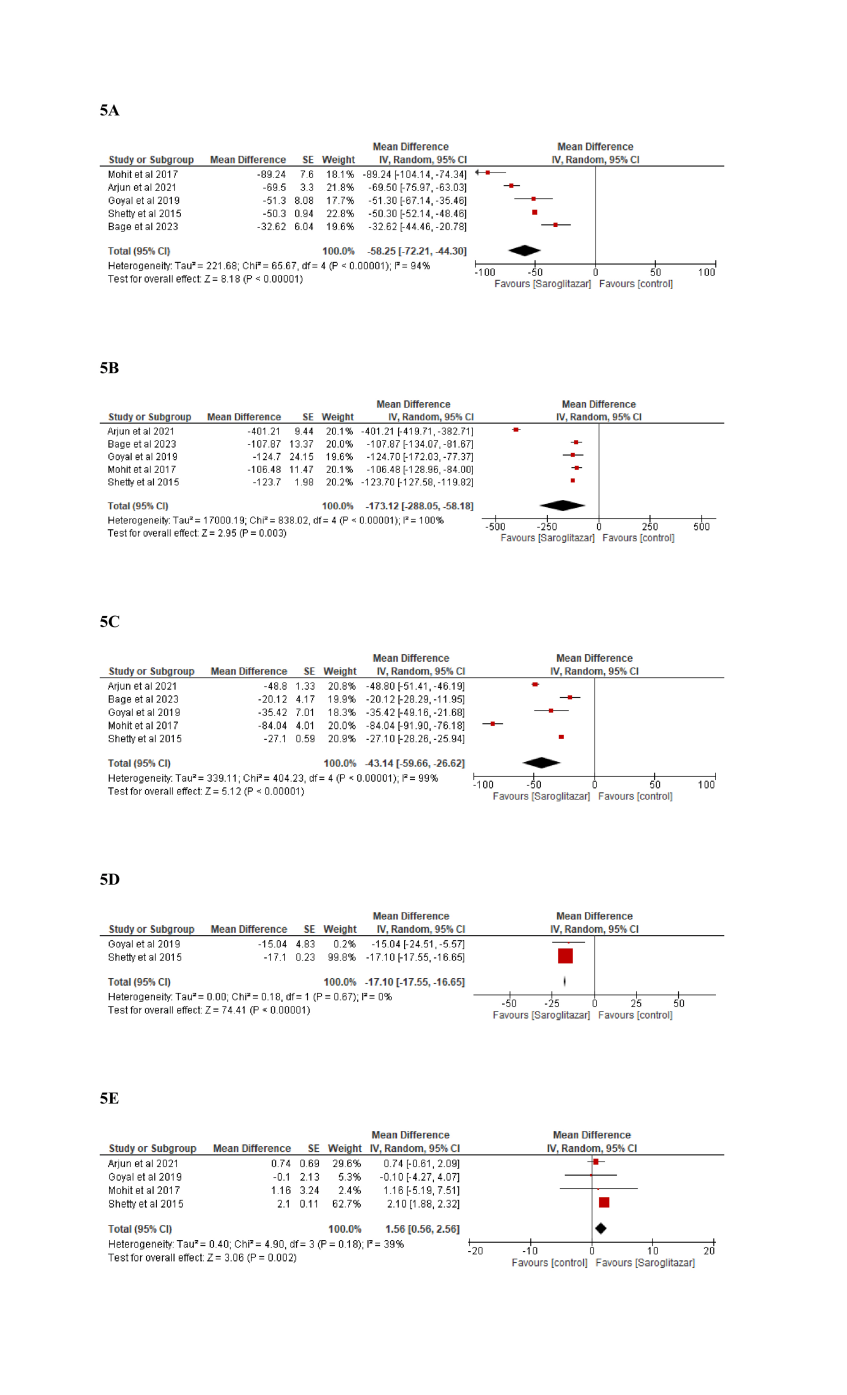
Forest plot showing reduction in lipid levels with saroglitazar 4 mg for the treatment of diabetic dyslipidemia (observational studies) Figure 5a: Total cholesterol [11-15] Figure 5b: Triglycerides [11-15] Figure 5c: LDL [11-15] Figure 5d: VLDL [13,15] Figure 5e: HDL [11,13-15] HDL: High-density lipoprotein; LDL: Low-density lipoprotein; VLDL: Very-low-density lipoprotein.

As compared to the control group, treatment with saroglitazar led to a significant reduction in LDL levels of -19.29 (95% CI: -29.78, -8.79) p<0.001, I^2^=95% in clinical trials and -43.14 (95% CI: -59.66, -26.62) p<0.001, I^2^=99% in observational studies. Pooled analysis of clinical trials has shown that saroglitazar significantly reduces VLDL levels by -16.9 (95% CI: -25.30, -8.49) p<0.001, I^2^=96%. Pooled analysis of the two observational studies has shown a statistically significant reduction in VLDL by -17.10 (95% CI: -17.55, -16.65) p<0.001, I^2^=0%. In addition, pooled analysis of clinical trials and observational studies has shown that saroglitazar improves levels of HDL by 2.43 (95% CI: 1.17, 3.70) p<0.001, I^2^=75% and 1.56 (95% CI: 0.56, 2.56) p=0.002, I^2^=39%, respectively. There was also a reduction in triglycerides by -90.91 (95% CI: -123.47, -58.34) p<0.001, I^2^ =95% and -173.52 (95% CI: -288.05, -58.18) p=0.003, I^2^ =100% in the pooled analysis of clinical trials and observational studies, respectively (Figures 4, 5). 

The safety parameters of the drug saroglitazar were recorded, and the adverse as well as serious adverse events (SAEs) are shown in Table 7. 

**Table 7:** Adverse events and serious adverse events AE: Adverse event; DSMB: Data Safety Monitoring Board; ECG: Electrocardiogram; SAE: Serious adverse event.

Study no.	Author, year	Drug administered	Duration of treatment (days)	Adverse events, no. of patients (%)	Name of adverse event(s)	Serious adverse events, no. of patients (%)	Name of serious adverse event(s)
Interventional studies							
1	Bhosle et al., 2018 [22]	Saroglitazar 4 mg (n=40)	168	1 (2.5)	One episode of diarrhea (resolved with antimicrobials, and the study drug was not stopped)	0	Nil
2.	Gahlot et al., 2023 [21]	Saroglitazar 4 mg (n=20)	84	3 (15)	Body ache, gastritis, and weakness	0	Nil
		Atorvastatin 10 mg (n=20)	84	6(30)	Bodyache, gastritis, weakness, nausea	0	Nil
3	Pai et al., 2014 [19]	Saroglitazar 2 mg (n= 41)	168	7 (17.07)	Asthenia, gastritis, chest discomfort, peripheral edema, dizziness, and tremors. Most adverse events were considered unrelated to treatment and were of mild intensity.	0	Nil
		Saroglitazar 4 mg (n=41)		7 (17.07)			
		Pioglitazone 45 mg (n=40)		11 (27.5)	Asthenia, gastritis, chest discomfort, weight gain, peripheral edema, dizziness, and tremors	2 (0.05)	One had suspected acute myocardial infarction (died) and another had hematemesis (hospitalized, treated, and discharged without any other sequelae). The investigators and DSMB have adjudicated these SAEs as non-treatment emergent.
4	Jani et al, 2014 [18]	Saroglitazar 2 mg (n=101)	84	6 (5.94)	Dyspepsia, gastritis, chest pain, pain, and pyrexia	2 (0.66)	There were two reports of hospitalization during this study due to chest pain that were classified as serious AEs. One subject was admitted and found to have a normal ECG, whereas another subject underwent coronary artery bypass grafting and informed the investigator subsequently. Both events were resolved and considered unrelated to the study drug. It is not mentioned the two patients belonged to which treatment arm.
		Saroglitazar 4 mg (n=99)		8 (8.08)			
		Placebo (n=102)		4 (3.92)	Gastritis, chest pain, and pain		
5	Jain et al., 2019 [17]	Saroglitazar 4 mg (N=15)	112	0	Nil	0	Nil
		Placebo (N=15)					
6	Rastogi et al., 2020 [20]	Saroglitazar 4 mg (N=15)	84	1 (6.67)	Mild rash (resolved completely). On blinded causality assessment, the rash was considered remotely related to saroglitazar.	0	Nil
		Placebo (N=15)		1 (6.67)	Muscle spasm and constipation (mild AEs resolving completely, none serious). On blinded causality assessment, constipation and muscle spasm were deemed possibly related to placebo.		
7	Krishnappa et al., 2020 [5]	Saroglitazar 2 mg (N=380)	168	98 (25.79)	Pyrexia, headache, nasopharyngitis, pain, cough, and hyperchlorhydria	2 (0.17)	One was a case of acute coronary syndrome and the other was of a coronary artery disease. Detailed causality assessment was performed by the study investigators and both the SAEs were termed "not related" to the study drug by taking into account the pre-existing past medical history of the patients. It is not mentioned the two patients belonged to which treatment arm
		Saroglitazar 4 mg (N=386)		43 (11.14)	Headache, pyrexia, and nausea		
		Pioglitazone 30 mg (N=389)		66 (16.97)	Headache, pyrexia, diarrhea, asthenia, and pain		
Observational studies							
8	Goyal et al., 2019 [13]	Saroglitazar 4 mg (n=50)	84	NM	NM	0	Nil
9	Shetty et al., 2015 [15]	Saroglitazar 4 mg (n=3133)	84	NM	NM	0	Nil
10	Bage et al., 2023 [12]	Saroglitazar 4 mg (n=54)	84	8 (15%)	Chest discomfort, gastritis, abdominal pain, dizziness, reddish stool, and salty taste	0	Nil
11	Mohit et al., 2017 [14]	Saroglitazar 4 mg (25, excluding patients with CAD)	168	NM	NM	0	Nil
12	Baidya et al., 2021 [11]	Saroglitazar 4 mg (n=150)	84	15 (10%)	Gastrointestinal discomfort, asthenia, muscle pain, hypoglycemia, and weight gain	0	Nil

Discussion

Saroglitazar, being a dual PPAR α/γ agonist, was approved for the treatment of diabetic dyslipidemia and hypertriglyceridemia in patients with type 2 diabetes mellitus, not controlled by statins, as an add-on treatment and as a second-line therapy with metformin for metabolic dysfunction-associated steatotic liver disease in India [4]. For the treatment of primary biliary cholangitis, it has a United States Food and Drug Administration (US FDA) orphan medication and fast track designation status [23].

Our study has shown that saroglitazar caused a mean HbA_1c_ reduction of -1.06% and -0.82% in a pooled meta-analysis of observational studies and randomized clinical trials, respectively, among individuals with type 2 diabetes mellitus who were treated for dyslipidemia over a 12- to 24-week period. Our findings were different from the findings of meta-analyses by Menezes et al. and Dutta et al., where HbA_1c_ level did not show a significant decrease [6,24].

In our study, compared to placebo, saroglitazar 2 mg and 4 mg doses significantly reduced HbA_1c_ in patients with diabetic dyslipidemia. However, among the 2 mg and 4 mg doses of saroglitazar, there was no discernible difference in the lowering of HbA_1c_. This was similar to the findings of meta-analysis by Dutta et al [6]. In our meta-analysis, saroglitazar was found to be associated with a significant mean reduction in FBS of -26.74 mg/dL compared to the baseline. These findings were similar to the previous meta-analysis, which revealed reductions in FBS of -24.61 mg/dL and -23.07 mg/dL, respectively [6,24]. Out of 12 included studies, only 4 studies reported on PPBS [5,11,15,17].

In the present meta-analysis, saroglitazar was observed to reduce total cholesterol by -26.15 mg/dL and -58.25 mg/dL in clinical trials and observational studies, respectively. The reduction in the total cholesterol was similar to the results of the meta-analysis by Menezes et al. [24]. Conversely, in Dutta et al., the reduction in total cholesterol was not statistically significant [6]. In observational studies and clinical trials, the mean reduction in triglycerides was -173.12 mg/dL and -90.91 mg/dL, respectively. This was similar to the results by Menezes et al. [24]. However, it was contrary to the study by Dutta et al., which did not demonstrate a decrease in triglyceride levels [6].

In our meta-analysis, both clinical trials and observational studies showed a mean reduction in LDL of -19.29 mg/dL and -43.14 mg/dL, respectively, which was statistically and clinically significant. The outcomes were similar to those of the meta-analysis by Menezes et al., which demonstrated a statistically significant reduction in LDL levels [24]. Out of the included studies, six studies have reported the effect of saroglitazar on VLDL levels. The VLDL was reported to have decreased by -16.9 mg/dL, which was statistically significant compared to placebo.

According to our study, saroglitazar increased HDL levels by a mean of 2.43 mg/dL in clinical trials, which was statistically significant. It was in contrast to the findings by Dutta et al. and Menezes et al., which showed changes in HDL levels of 1.16 mg/dL and -1.38mg/dL, respectively [6,24].

Six SAEs were recorded, but none of the significant adverse events were linked to saroglitazar in terms of safety profile. In the study by Pai et al., one patient had a suspected acute myocardial infarction resulting in death and another had hematemesis without any other sequelae [19]. Both the above SAEs were adjudicated as non-treatment emergent. In the study by Jani et al., two SAEs requiring hospitalization due to chest pain were reported [18]. Both events were reported by the authors as unrelated to the study drug. Similarly, in the study by Krishnappa et al., one case of acute coronary syndrome and the other of coronary artery disease were reported, which turned out to be “not related” following causality assessment. The majority of the adverse effects were minor in nature. The most frequent adverse effects found in the studies were headache, pyrexia, gastritis, and discomfort.

Limitations

Most of the clinical trials included in the study had a smaller sample size. The duration of treatment also varied across the study and was shorter (84-168 days). Hence, the study results need to be generalized with caution. Further, the heterogeneity was high (>60%) in terms of efficacy results.

## Conclusions

Our meta-analysis has found that saroglitazar causes a significant reduction in the HbA_1c_, FBS, and PPBS levels in patients treated for diabetic dyslipidemia.
